# New technical approach for the repair of an abdominal wall defect after a transverse rectus abdominis myocutaneous flap: a case report

**DOI:** 10.1186/1752-1947-2-108

**Published:** 2008-04-16

**Authors:** Daniel A Kaemmer, Joachim Conze, Jens Otto, Volker Schumpelick

**Affiliations:** 1 Department of Surgery, Medical Faculty, Rheinish-Westphalian Technical University, D-52074 Aachen, Germany

## Abstract

**Introduction:**

Breast reconstruction with autologous tissue transfer is now a standard operation, but abnormalities of the abdominal wall contour represent a complication which has led surgeons to invent techniques to minimize the morbidity of the donor site.

**Case presentation:**

We report the case of a woman who had bilateral transverse rectus abdominis myocutaneous flap (TRAM-flap) breast reconstruction. The surgery led to the patient developing an enormous abdominal bulge that caused her disability in terms of abdominal wall and bowel function, pain and contour. In the absence of rectus muscle, the large defect was repaired using a combination of the abdominal wall component separation technique of Ramirez et al and additional mesh augmentation with a lightweight, large-pore polypropylene mesh (Ultrapro^®^).

**Conclusion:**

The procedure of Ramirez et al is helpful in achieving a tension-free closure of large defects in the anterior abdominal wall. The additional mesh augmentation allows reinforcement of the thinned lateral abdominal wall.

## Introduction

Abnormalities of the abdominal wall contour after breast reconstruction with autologous tissue transfer have previously been reported as problematic, with a lower abdominal bulge being the most frequently reported abnormality [1]. Although the cosmetic results and patient satisfaction seem to be good in most cases with regards to shape, symmetry and muscular function, differences become obvious in the morbidity of the donor site [2-5]. Modifications and new techniques have been developed to reduce complications, but none of these modifications is able to prevent contour abnormalities of the donor site completely [6], and new techniques, which preserve the anterior rectus sheath are limited in their use by anatomic variations [2].

In addition to the aesthetic disturbance, these defects can also lead to adverse interference of the abdominal wall functions, as a thrust bearing for the intraabdominal pressure and as an antagonist of the back muscles and part of the respiratory system. To date, these side effects have not attracted attention in the literature and no therapeutic approaches have been reported.

Here we present the case of a woman with an extreme bulge of the lower abdominal wall following bilateral transverse rectus abdominis myocutaneous flap (TRAM-flap) breast reconstruction. This was repaired using a combination of the abdominal wall component separation technique of Ramirez et al [7] and additional mesh augmentation.

## Case presentation

We report the case of a 61-year-old woman who was suffering from lower abdominal bulge formation, chronic constipation, as well as a feeling of permanent abdominal constriction and pain. These symptoms appeared eight months after bilateral breast reconstruction, which was performed following subcutaneous mastectomy that was necessary owing to ductal carcinoma *in situ*. The breast reconstruction was conducted using a non-muscle-sparing pedicled TRAM-flap transposition. The defect created at the donor site within the abdominal wall after harvesting the rectus muscle was closed using a continuous suture with resorbable suture material. An additional augmentation was performed by the implantation of a resorbable polyglactin mesh placed on the fascial suture.

**Figure 1:**
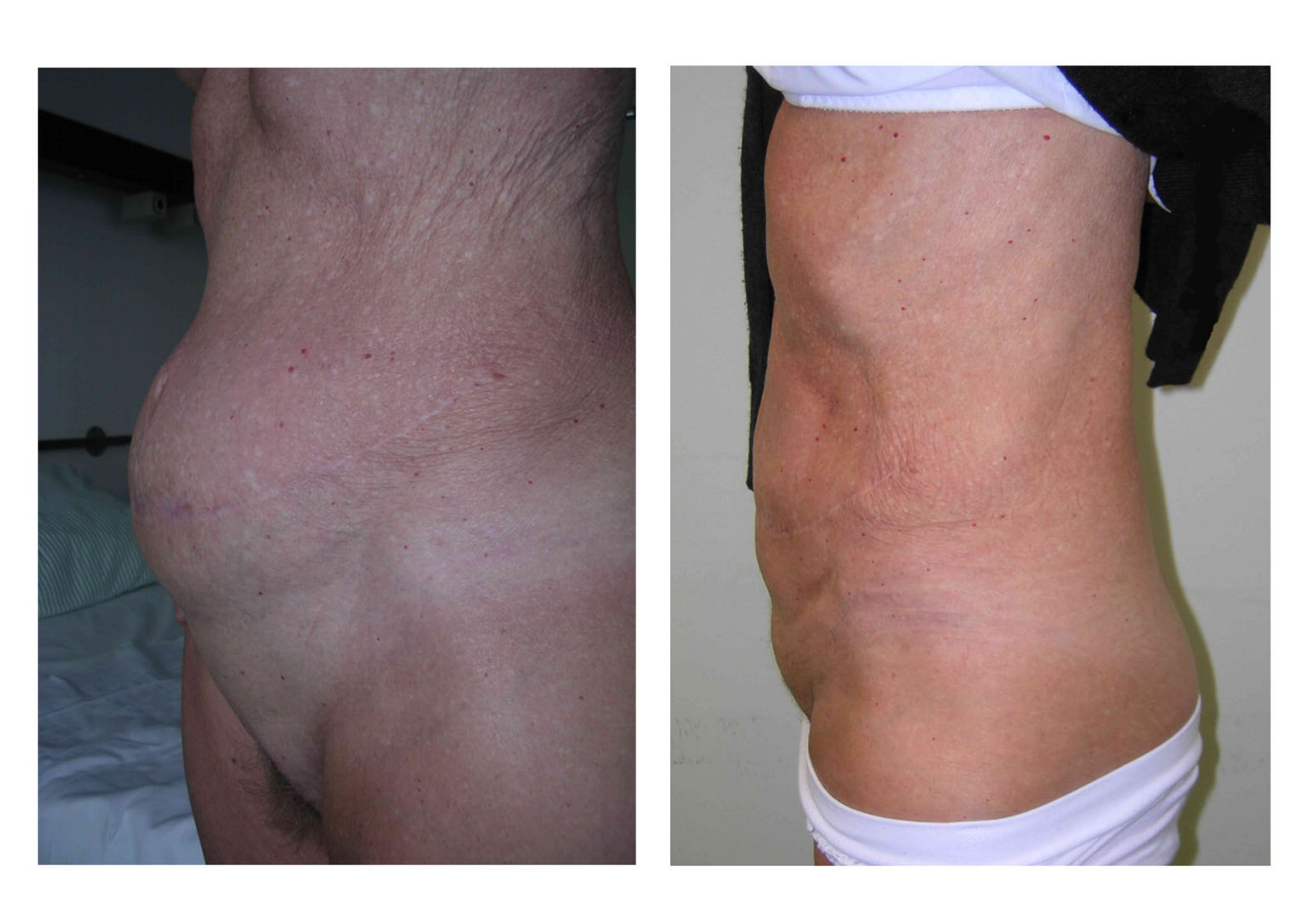
**Abdominal contour before and after reconstruction**. (A) The preoperative abdominal contour (lateral view). (B) The abdominal contour six weeks after the reconstruction (lateral view). In addition to minimizing the abdominal bulge, Ramirez et al's technique is able to shape the lateral abdominal wall in an aesthetic manner; lateral bulging was avoided using mesh augmentation.

The patient presented at the authors' outpatient clinic eight months after reconstruction. At that time her body mass index was 18.9 and she was suffering from a lower abdominal bulge formation (Figure 1). An ultrasound examination revealed an abdominal wall defect measuring 18 × 20 cm, with no detectable rectus abdominis muscle remaining, resembling a large rectus diastasis. A preoperative endoscopy of the colon showed signs of adhesions in the colon sigmoideum and transversum, but no other pathologies; the laboratory values were normal. Apart from an appendectomy performed 20 years ago, the patient had undergone no other previous abdominal surgery. In addition to the annoying large bulge in this otherwise slim patient, the pain experienced during everyday movement and impairment of bowel function led to an explorative laparotomy and an attempt to reconstruct the abdominal wall.

**Figure 2:**
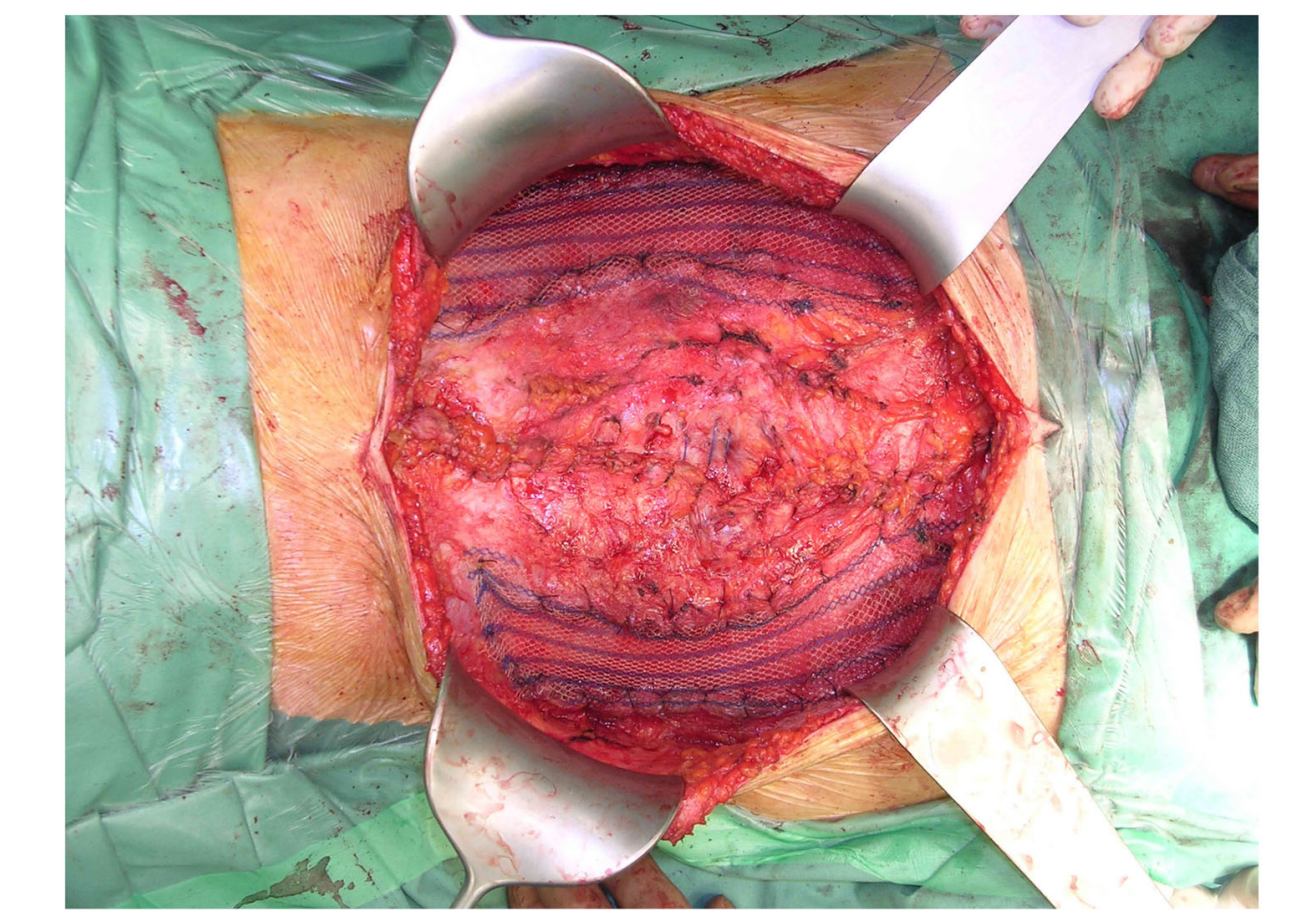
**Mesh augmentation using two halfmoon-shaped lightweight polypropylene meshes placed on the defects between the external oblique muscles and lineae semilunares**. The meshes were fixed using resorbable single-stitch sutures. After a midline incision and adhesiolysis, the abdominal wall components were separated along the avascular plane between the internal and external oblique abdominal muscles. A midline closure in two layers was performed using non-resorbable single-stitch sutures and continuous slowly resorbable suture for the posterior wall and anterior rectus sheath, respectively.

Following adequate preparations with intestinal irrigation, a re-incision through the midline scar was performed. On entering the peritoneal cavity, several dense adhesions of small intestine to the abdominal wall and interenteric to the colon were found. These were carefully dissolved without causing injury to the intestine. Further exploration revealed a near-total absence of both abdominal rectus muscles; residual muscle fibres could be detected only at the lateral side of the rectus sheath. The initially implanted absorbable mesh was not identified, and the ultrasonographic finding of a diastasis-like defect with lateralization of both lineae semilunares was verified. Following a wide-ranging mobilization of the epifascial subcutaneous tissue, the remaining parts of the anterior rectus sheaths and minimal lateral parts of the rectus muscles were exposed. The herniation sac was partly resected, leaving sufficient material to facilitate a peritoneal closure of the abdominal cavity. In order to reach an adaptation of both lateralized anterior rectus sheaths, a component separation of the abdominal wall (Ramirez procedure) was performed. In the absence of an intact rectus abdominis muscle and anterior rectus sheath, only a vertical incision lateral to the linea semilunaris and separation in the plane between oblique external and internal muscle was used. A two-layer closure of the fascia in the midline was performed using a non-resorbable single-stitch suture of the posterior wall, and a continuous suture with a slowly resorbable suture material for the remaining anterior rectus sheath. The lateral defects between the external oblique muscle and linea semilunaris were covered with a halfmoon-shaped lightweight polypropylene mesh (Ultrapro^®^; Ethicon, Norderstedt, Germany) on each side (Figure 2). Punctual mesh fixation was achieved using resorbable 3/0 single-stitch sutures (Dexon^®^; Braun, Germany). A subcutaneous suction drain was placed on top of each mesh, after which wound closure was achieved with a continuous intracutaneous suture using non-resorbable material.

The patient's recovery was uneventful; during her hospital stay she wore an elastic abdominal belt and was provided with analgesics and physical therapy with intense respiratory training. The suction drains and suture material were removed on schedule, the postoperative ultrasonography was without pathological findings and minimal postoperative seroma resolved. The patient was discharged from hospital and made subsequent visits to the outpatient clinic. At 12 months after surgery she remained satisfied with the outcome.

## Discussion

The TRAM-flap technique developed by Hartrampf et al [8] in 1982 is now well established. Long-term evaluations of any complications and aesthetic outcome have been conducted which state that, for the TRAM-flap, the rate of ('true') hernia or abdominal bulge is about 0–5% [5]. Modifications of the original technique have been developed, including muscle- and fascia-sparing techniques [9] as well as free flaps [10] and mesh implantation [11]. These modifications have reduced the incidence of complications, such as hernia and bulge formation, in the remaining abdominal wall.

The anterior rectus sheath is one of the major components maintaining the integrity of the abdominal wall and contour; consequently, flaps which preserve this structure completely have been evaluated [12]. The deep inferior epigastric perforator flap (DIEP-flap) is an alternative, widely used modification, and surgeons have also described and used the superficial inferior epigastric artery flap (SIEA-flap) or gluteal artery perforator flap (GAP-flap) [13]. These flaps preserve the anterior rectus sheath and therefore minimize the risk of a hernia or bulge formation, although this has been described in the case of DIEP-flaps and is considered to be a result of denervation. The myocutaneous flap has no advantages in terms of autologous tissue volume and the possibility of modelling symmetric and natural-looking breasts. SIEA-flaps can only be used if a superficial inferior epigastric artery is present and is sufficient to perfuse the flap, but in this select patient group it may be used as the first choice [2]. Today, GAP-flaps are considered as a fall-back technique and are used only if abdominal cutaneous tissue and fat is not appropriate for the reconstruction.

**Figure 3:**
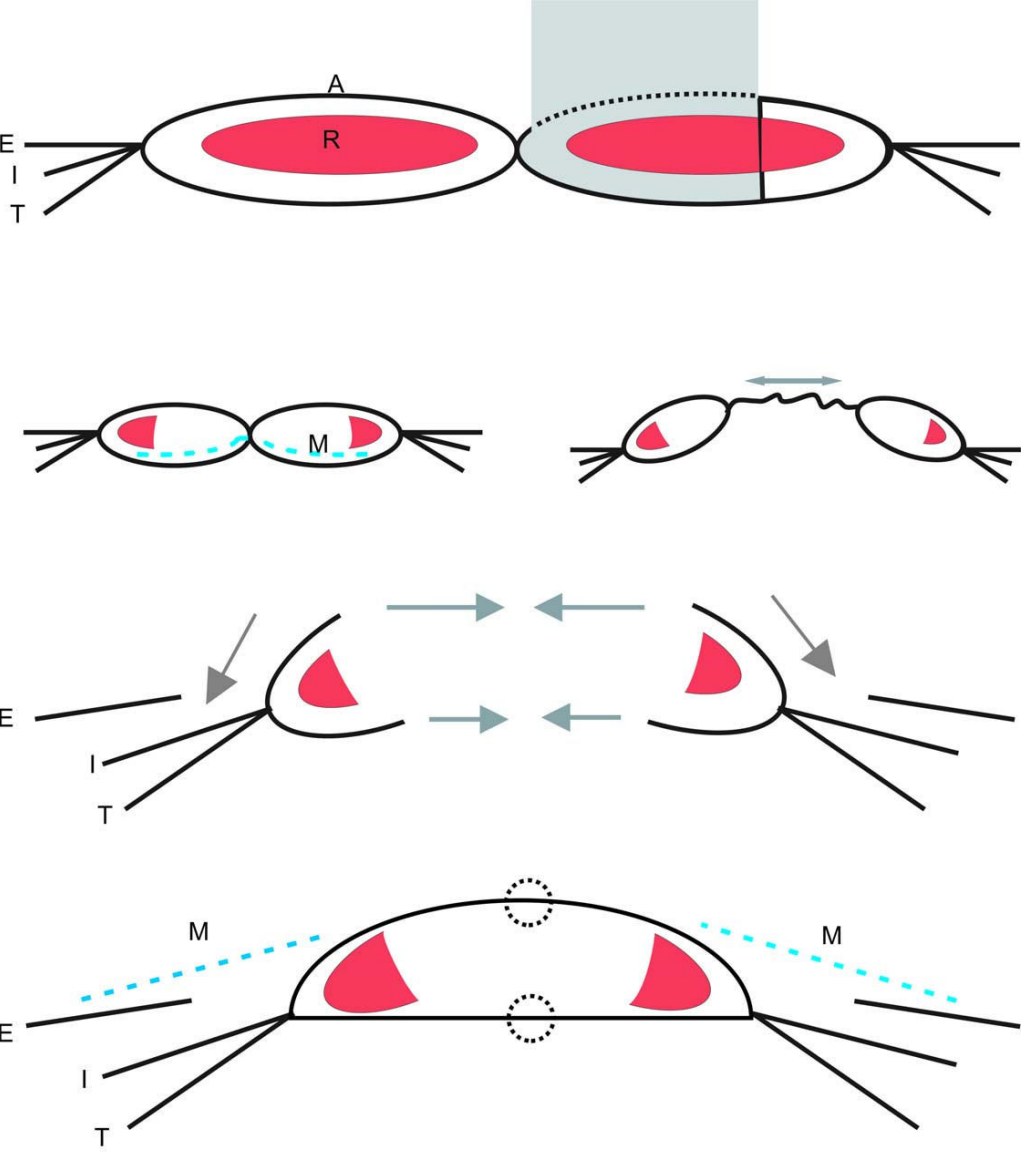
**Schema of the abdominal wall**. (A) The normal abdominal wall. (B) Left: postoperative conditions after bilateral TRAM-flap. Right: abdominal bulge that developed in the present case. (C) Conditions after abdominal wall component separation, before double-layer midline closure. (D) Postoperative conditions after mesh augmentation.

In the case described in this report the bilateral non-muscle-sparing TRAM-flap transfer led to an enormous abdominal bulge that caused disability for the patient in many different ways. To date, no standard surgical procedure has been developed to treat these defects. Damage to the TRAM-flap resulted in a broad defect in the area of the harvested rectus muscle that could not be reversed (Figure 3). The principal idea of any repair should be to reconstruct the abdominal wall integrity with closure of the fascial defect. In 1990, Ramirez et al [7] described a component separation technique which allowed a midline advancement of the abdominal wall of up to 10 cm on each side, without the need for musculofascial flaps. Moreover, this technique provides an innervated and vascularized compound for dynamic support by dividing the abdominal wall components along an avascular plane.

Additional mesh augmentation was not used in the original component separation method described by Ramirez et al. The anterior rectus sheath was opened and the rectus muscle was separated from the posterior rectus sheath and moved medially. In the present case, because there was almost no rectus muscle remaining, it was necessary to omit this step. A longitudinal incision was made lateral to the border of the rectus sheath and separation continued in the more-or-less avascular plane between the external and internal oblique muscles, leaving the external oblique lateral to the subsequently closed midline incision. To the best of the authors' knowledge, the component separation technique has been performed previously with only one rectus muscle remaining, but never without any rectus muscle on either side. In contrast, it was stated that at least one innervated rectus was required to re-establish the integrity of the abdominal wall [7].

The idea of mesh augmentation in the midline was abandoned owing to the fact that, in the present patient, there was no typical incisional hernia pathophysiology but rather an abdominal wall defect that had been created deliberately, and this made a collagen defect unlikely. The use of mesh material was reduced to only augmenting the thinned lateral abdominal wall, to prevent any possible postoperative bulging of the internal oblique and transverse muscles. For the same contouring reasons, and to avoid extensive adhesion formation, a mesh prosthesis placed intraperitoneally using an onlay technique (IPOM) [14] was not used. Furthermore, this technique would have required replacement rather than augmentation of the abdominal wall.

An extensive epifascial preparation might put the blood circulation of the skin at risk. In slim patients, where the subcutaneous layer is not usually pronounced, the additional use of excessive foreign material should be considered carefully. The use of lightweight, large-pore polypropylene meshes appears to reduce the risk of any major foreign-body reaction that might lead to shrinkage of the mesh area or to a reduction in abdominal wall mobility [15]. The textile features of this new mesh generation are more adapted to the physiology of the abdominal wall and are predisposed to its augmentation [16].

## Conclusion

It has been shown that a reconstruction of the abdominal wall midline is possible and maintainable in the absence of both rectus muscles, using the component separation technique of Ramirez et al. A modification is suggested using additional mesh augmentation to cover the thinned lateral abdominal wall, using a lightweight polypropylene mesh prosthesis.

## Competing interests

The author(s) declare that they have no competing interests.

## Authors' contributions

DAK assisted with the surgery, designed the case report, collated the information, performed the literature search and prepared the manuscript. JC assisted with the surgery, was involved in all investigations and assisted in providing a critical appraisal and review of the manuscript. JO prepared the images, advised on the format and design and assisted in providing a critical appraisal of the manuscript. VS performed the surgery, was involved in all investigations and assisted in the literature search, writing and editing of the manuscript. All authors have reviewed and approved the final manuscript.

## Consent

Written informed consent was obtained from the patient for publication of this case report and accompanying images. A copy of the written consent is available for review by the Editor-in-Chief of this journal.
